# Correction: Both conventionally and organically fertilized tomatoes maintain fruit quality through uncontrolled green peach aphid infestation, with a transcriptional shift towards catabolism

**DOI:** 10.1371/journal.pone.0356650

**Published:** 2026-08-19

**Authors:** June Labbancz, Luke Gustafson, Preston Andrews, Amit Dhingra

The figure captions are align properly, but the images are in the wrong order. The authors have provided a corrected version of the figures here (Fig 1–11).

**Fig 1:**
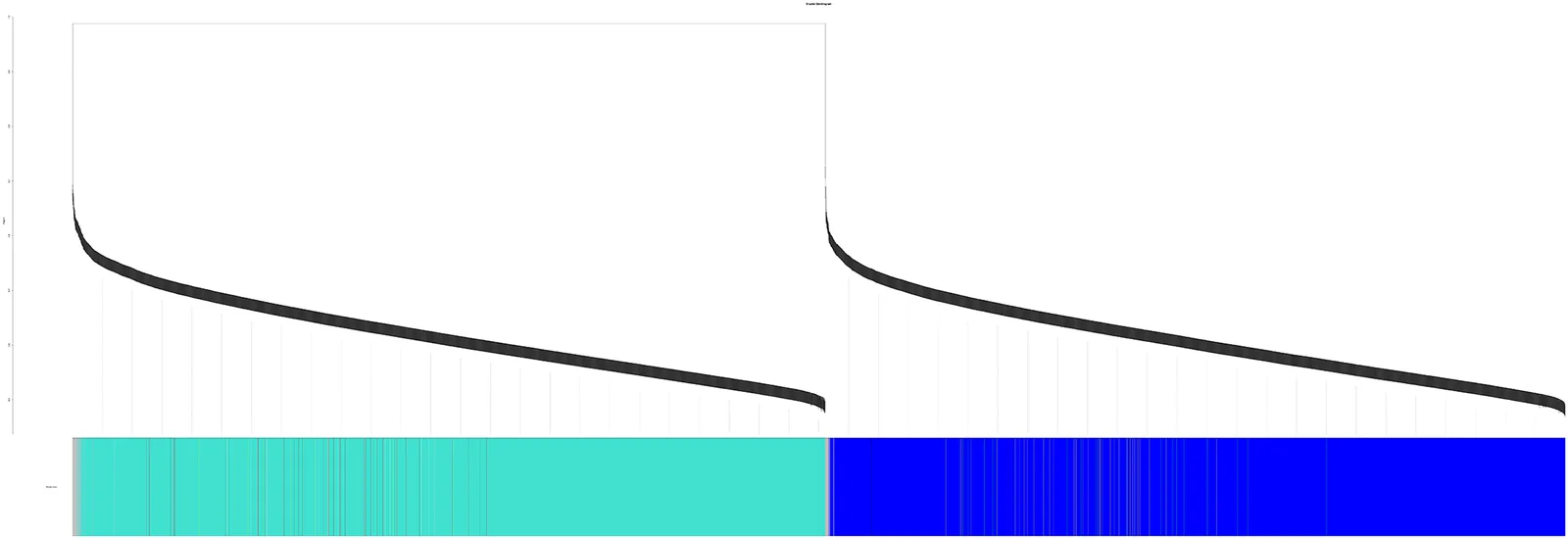
Cluster dendrogram describing the gene co-expression network generated from leaf samples in this study. Modules were identified using the dynamic tree cutting algorithm with a minimum module size of 30 genes and a merge cut height of 0.25.

**Fig 2:**
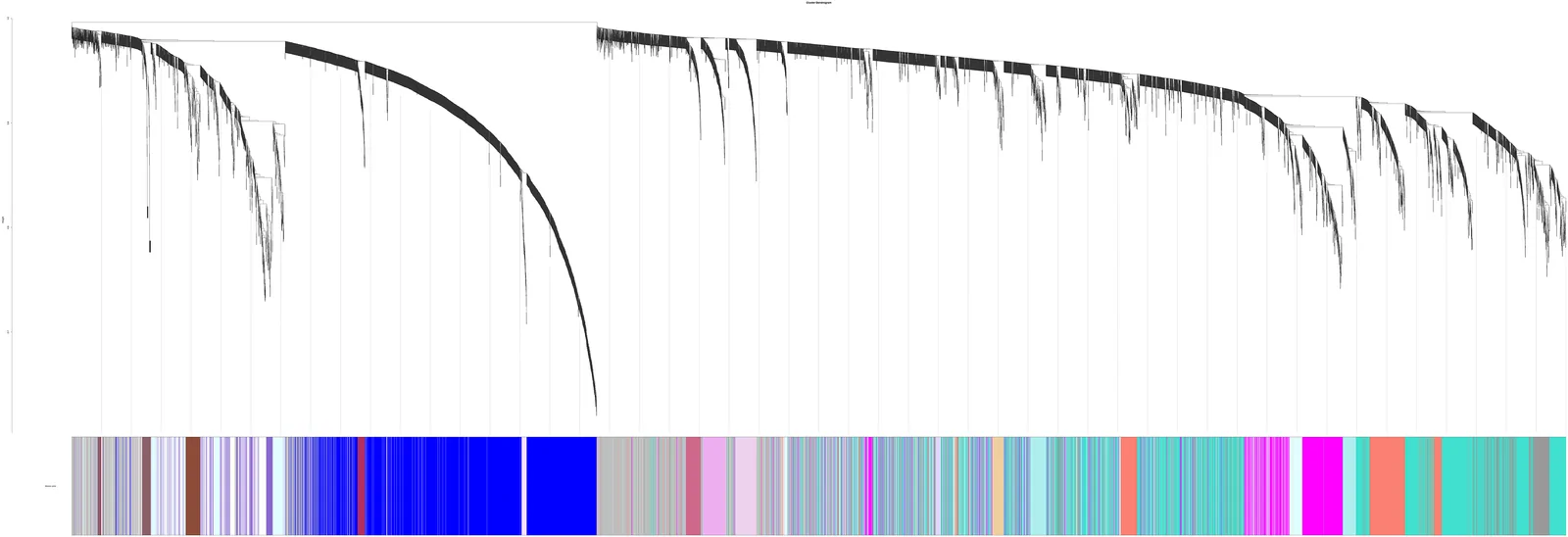
Cluster dendrogram describing the gene co-expression network generated from fruit samples in this study. Modules were identified using the dynamic tree cutting algorithm with a minimum module size of 30 genes and a merge cut height of 0.25.

**Fig 3:**
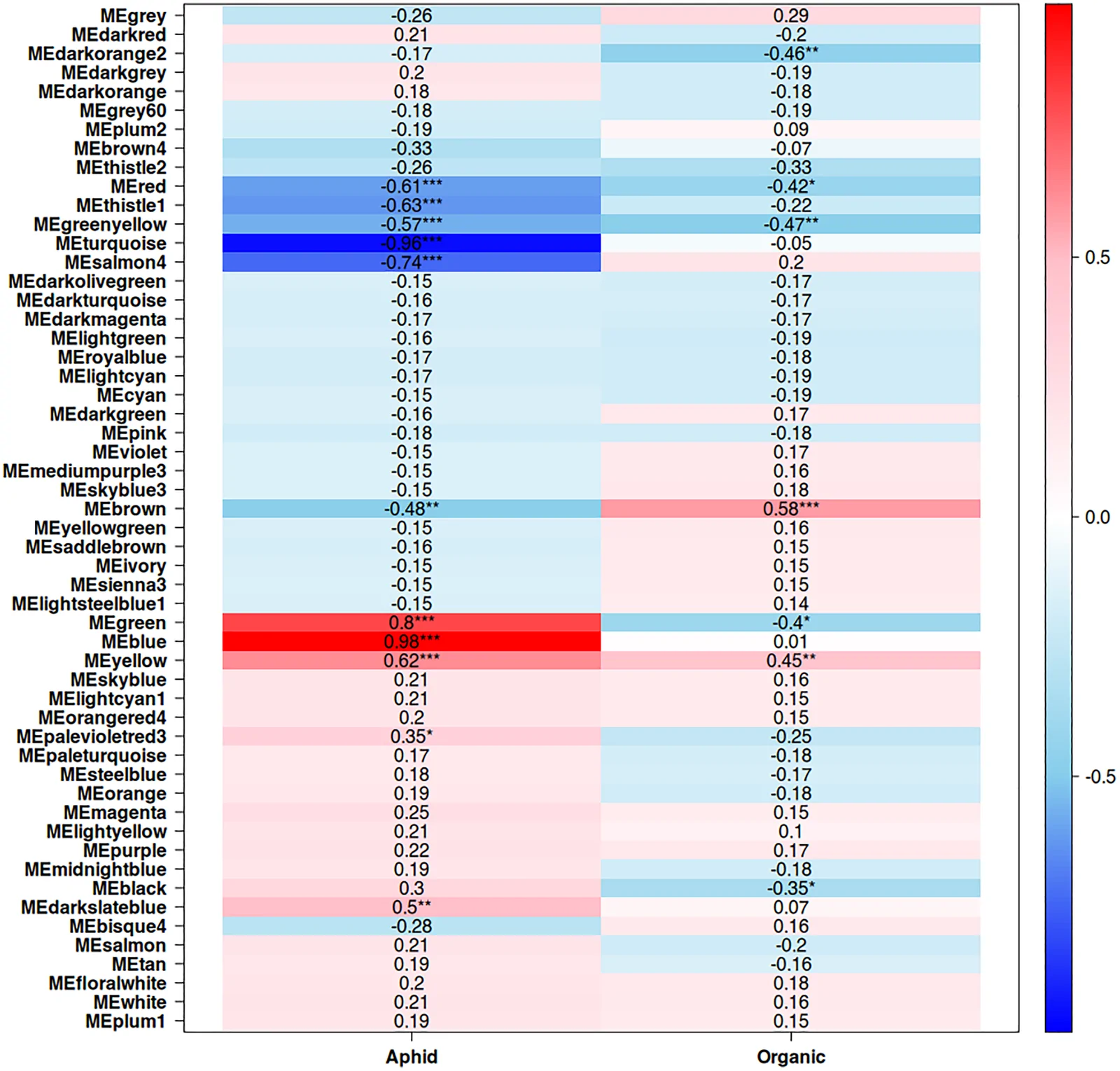
Correlations between modules and aphid presence and organic fertilizer treatment in the gene co-expression network generated from leaf samples. * = p < 0.5, ** = p < 0.1, *** = p < 0.01.

**Fig 4:**
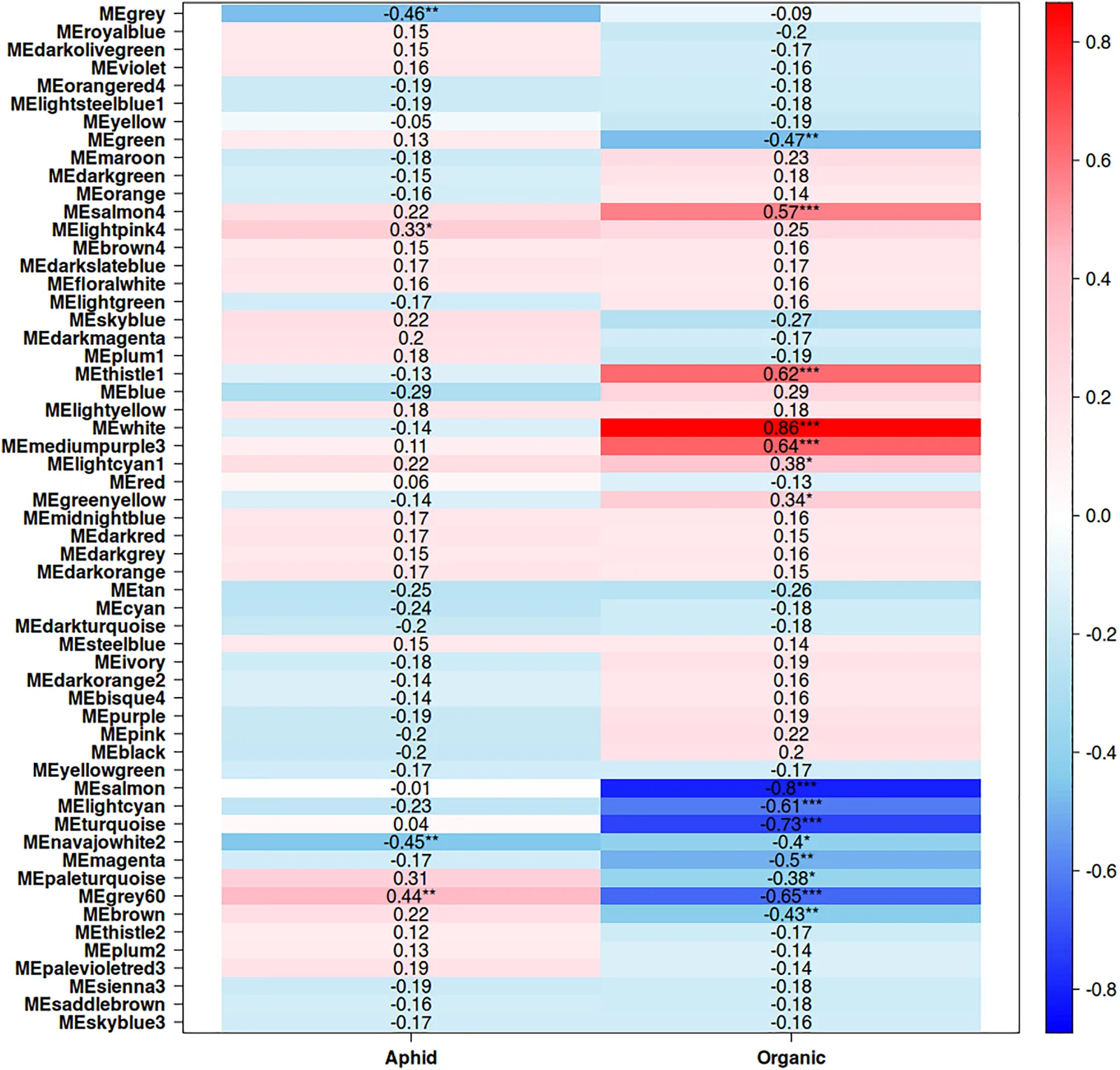
Correlations between modules and aphid presence and organic fertilizer treatment gene co-expression network generated from fruit samples. * = p < 0.5, ** = p < 0.1, *** = p < 0.01.

**Fig 5:**
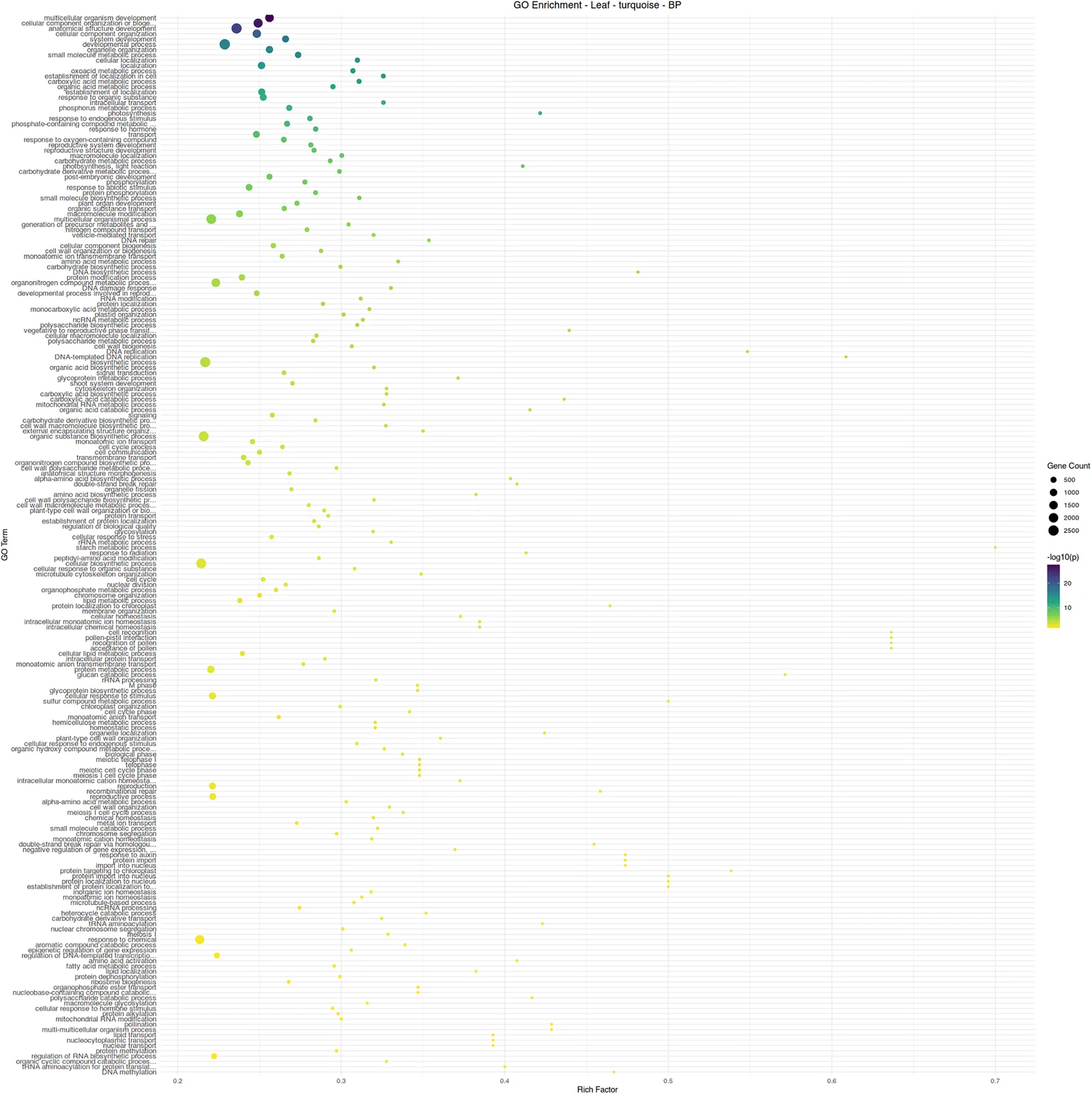
Gene ontology term enrichment analysis by Fisher’s exact test for biological processes in the leaf module turquoise, associated with aphid infestation. Significance is shown by circle color (scale bar to the right), number of genes are shown by circle size, and rich factor (proportion of genes in the module relative to genes in the annotated dataset) is plotted along the x-axis.

**Fig 6:**
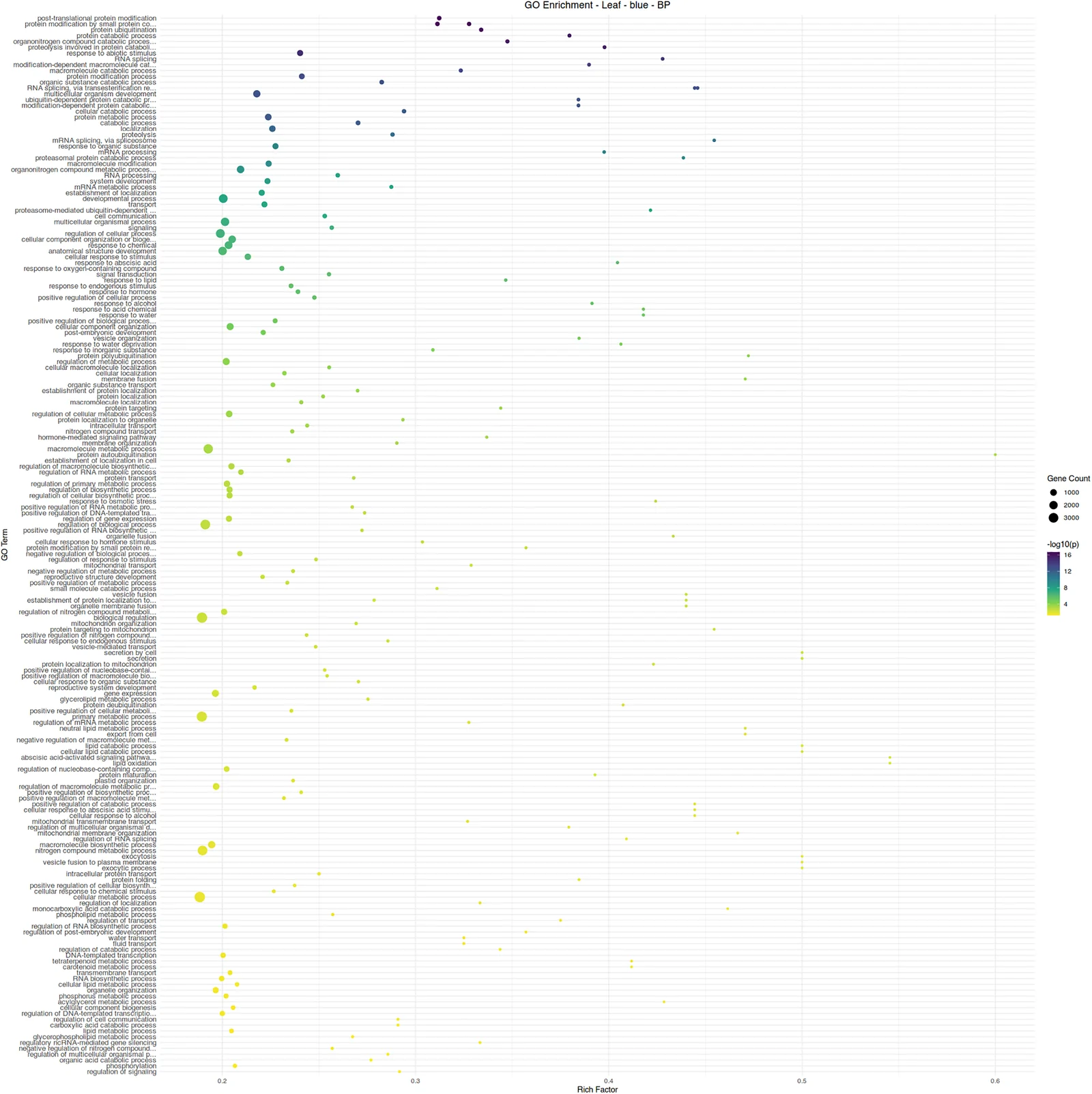
Gene ontology term enrichment analysis by Fisher’s exact test for biological processes in the leaf module blue, associated with the absence of aphid infestation. Significance is shown by circle color (scale bar to the right), number of genes are shown by circle size, and rich factor (proportion of genes in the module relative to genes in the annotated dataset) is plotted along the x-axis.

**Fig 7:**
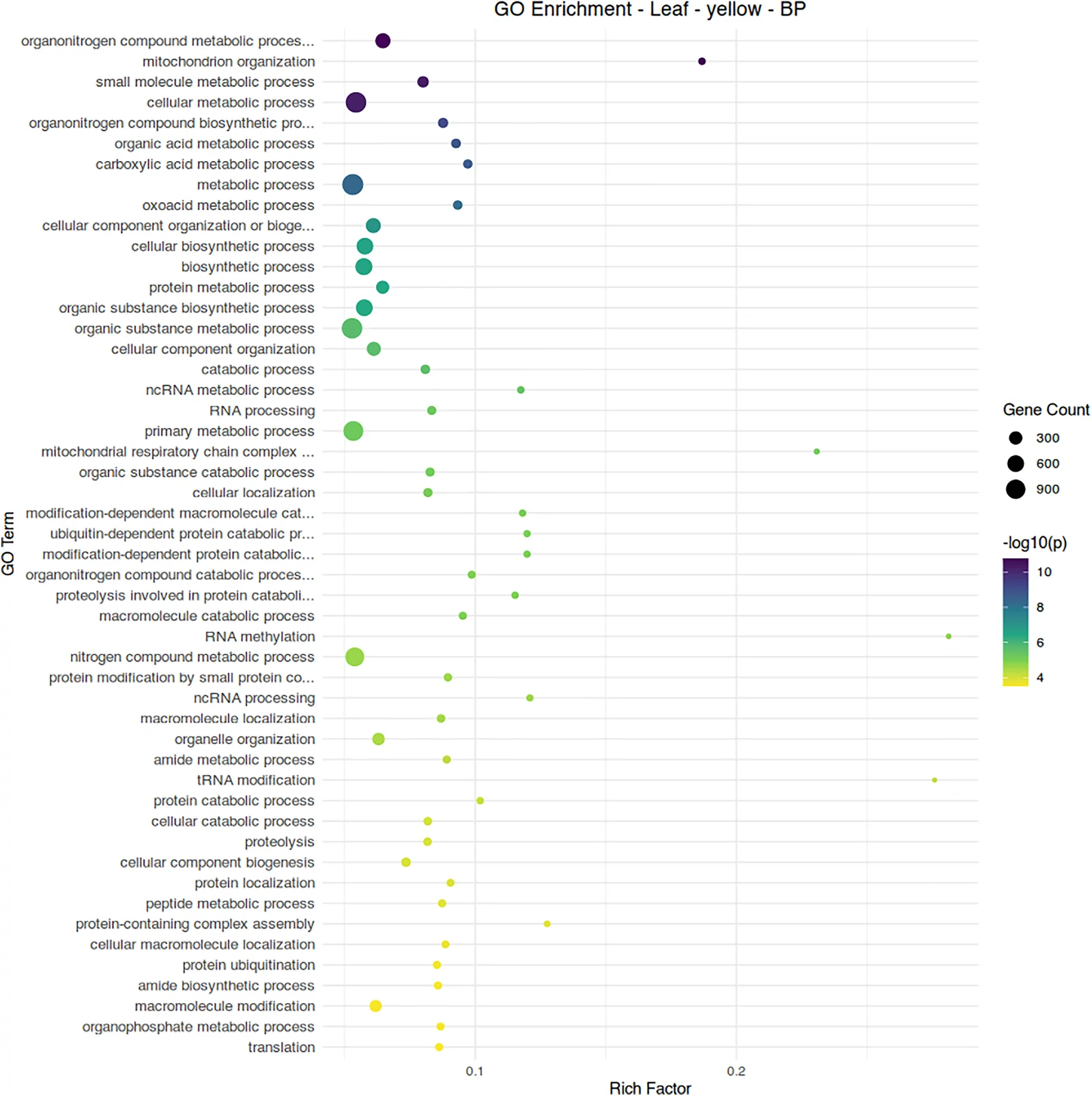
Gene ontology term enrichment analysis for biological processes in the leaf module yellow, associated with aphid infestation and organic fertilizer treatment. Significance is shown by circle color (scale bar to the right), number of genes are shown by circle size, and rich factor (proportion of genes in the module relative to genes in the annotated dataset) is plotted along the x-axis.

**Fig 8:**
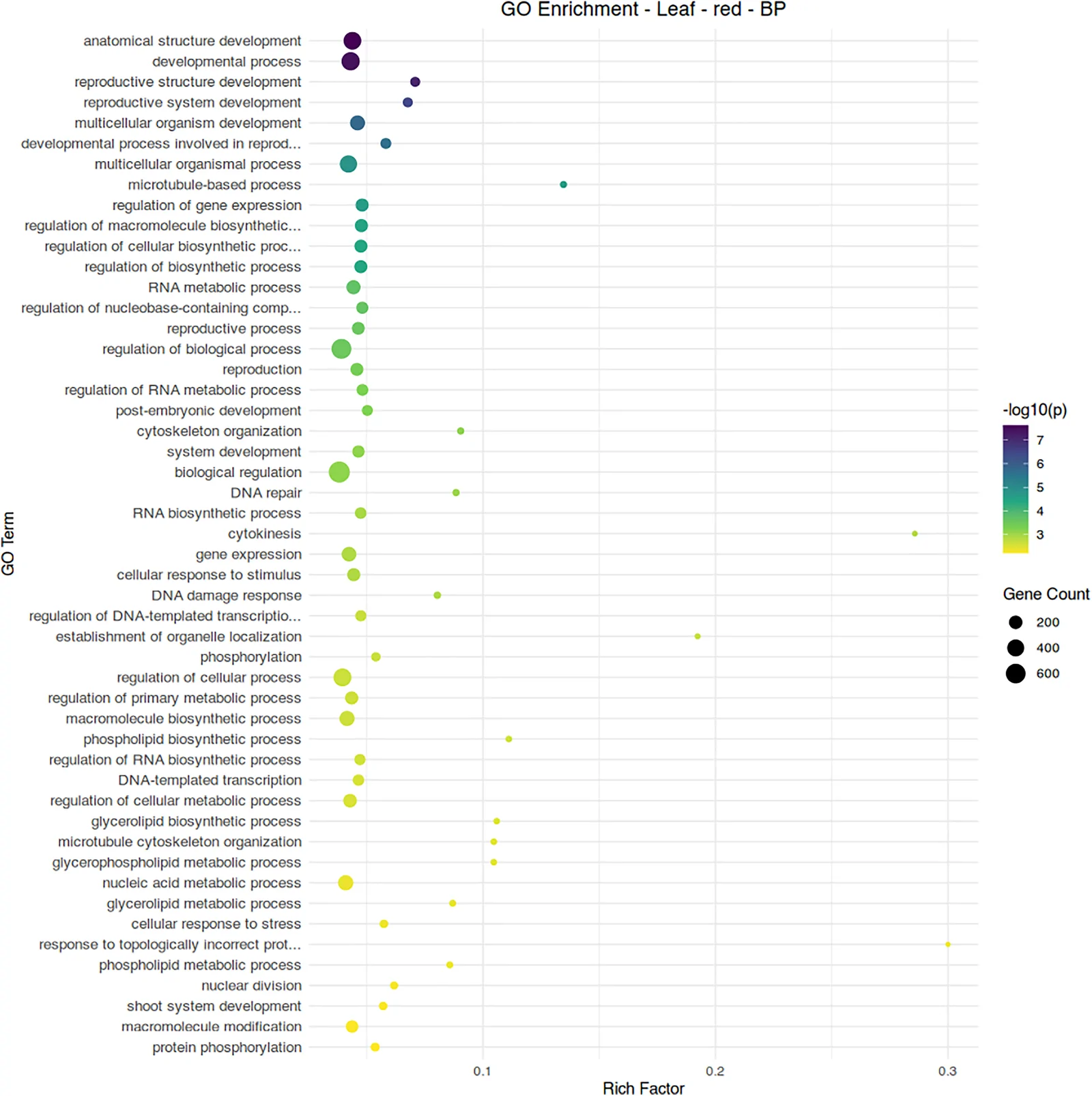
Gene ontology term enrichment analysis by Fisher’s exact test for biological processes in the leaf module red, associated with conventional fertilizer treatment and the absence of aphid infestation. Significance is shown by circle color (scale bar to the right), number of genes are shown by circle size, and rich factor (proportion of genes in the module relative to genes in the annotated dataset) is plotted along the x-axis.

**Fig 9:**
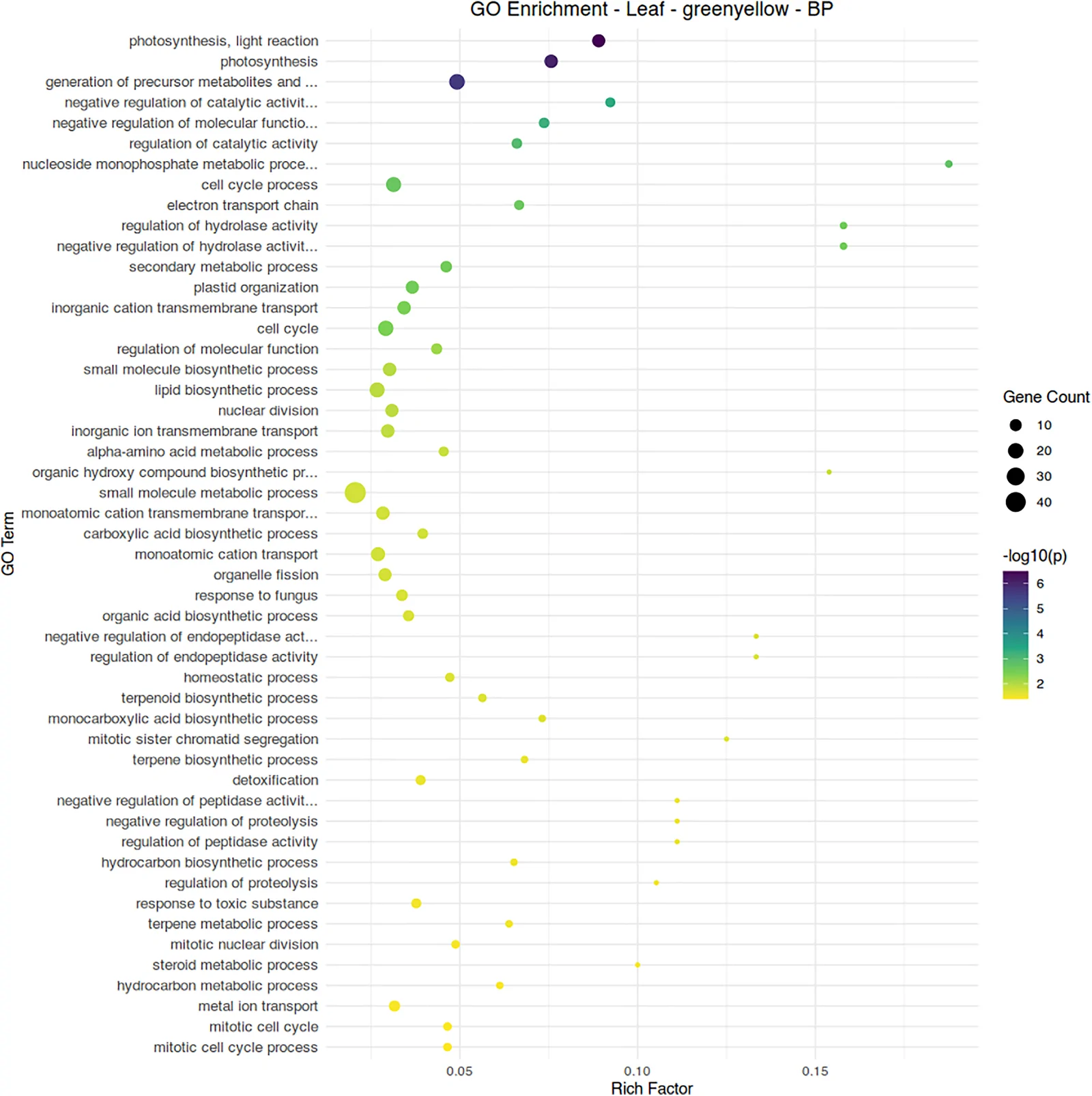
Gene ontology term enrichment analysis by Fisher’s exact test for biological processes in the leaf module greenyellow, associated with conventional fertilizer treatment and the absence of aphid infestation. Significance is shown by circle color (scale bar to the right), number of genes are shown by circle size, and rich factor (proportion of genes in the module relative to genes in the annotated dataset) is plotted along the x-axis.

**Fig 10:**
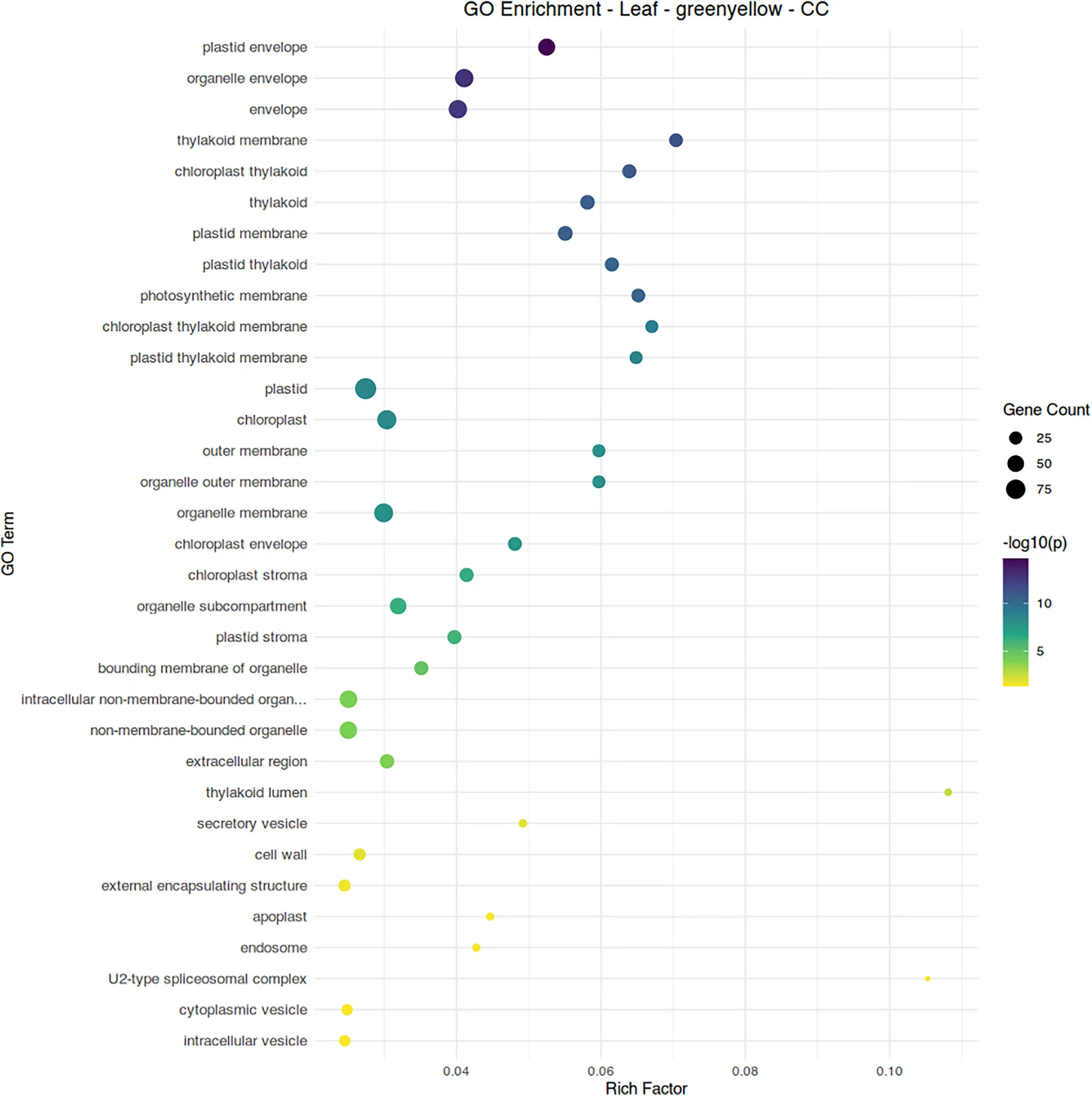
Gene ontology term enrichment analysis by Fisher’s exact test for subcellular localization in the leaf module greenyellow, associated with conventional fertilizer treatment and the absence of aphid infestation. Significance is shown by circle color (scale bar to the right), number of genes are shown by circle size, and rich factor (proportion of genes in the module relative to genes in the annotated dataset) is plotted along the x-axis.

**Fig 11:**
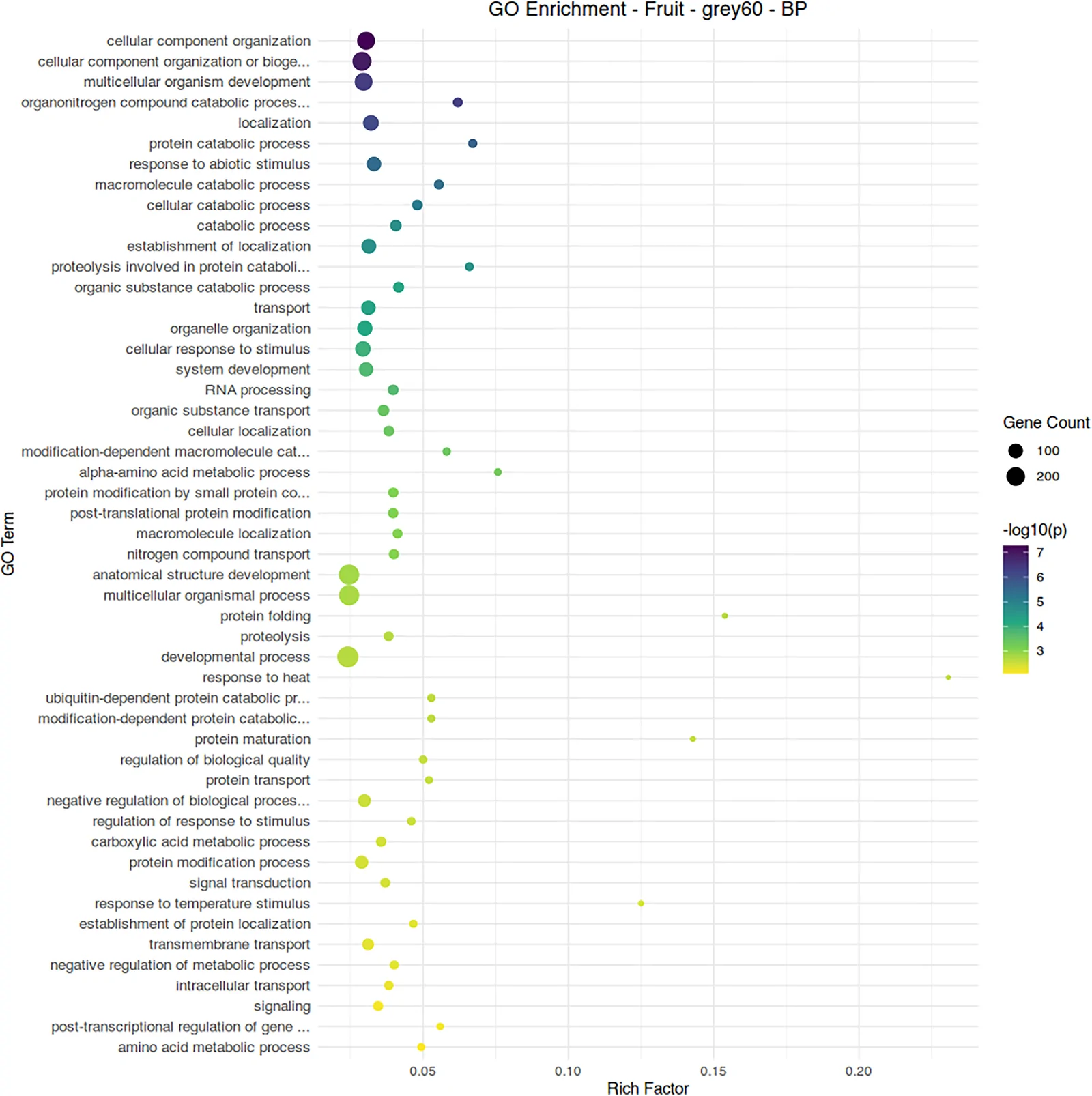
Gene ontology term enrichment analysis by Fisher’s exact test for biological processes in the fruit module grey60, associated with aphid infestation and conventional fertilizer. Significance is shown by circle color (scale bar to the right), number of genes are shown by circle size, and rich factor (proportion of genes in the module relative to genes in the annotated dataset) is plotted along the x-axis.
